# Effect of the Addition of Alginate and/or Tetracycline on Brushite Cement Properties

**DOI:** 10.3390/molecules26113272

**Published:** 2021-05-28

**Authors:** Claudia Morilla, Elianis Perdomo, Ana Karla Hernández, Ramcy Regalado, Amisel Almirall, Gastón Fuentes, Yaima Campos Mora, Timo Schomann, Alan Chan, Luis J. Cruz

**Affiliations:** 1 Biomaterials Center, University of Havana, La Habana 10400, Cuba; C.Morilla_Espino@lumc.nl (C.M.); anakarla@estudiantes.fq.uh.cu (A.K.H.); ramcy@estudiantes.fq.uh.cu (R.R.); amisel@biomat.uh.cu (A.A.); mana@biomat.uh.cu (Y.C.M.); 2 Translational Nanobiomaterials and Imaging Group, Department of Radiology, Leiden University Medical Center, 2333 ZA Leiden, The Netherlands; T.Schomann@lumc.nl (T.S.); L.J.Cruz_Ricondo@lumc.nl (L.J.C.); 3 Percuros B.V., 2333 CL Leiden, The Netherlands; achan@percuros.com; 4 Faculty of Automatic and Biomedical Engineering, Technological University of Havana, La Habana 11300, Cuba; elianisperdomo@gmail.com

**Keywords:** brushite, calcium phosphate cements, physical-chemical properties, mechanical properties, microbiological properties, cell viability

## Abstract

Calcium phosphate cements have the advantage that they can be prepared as a paste that sets in a few minutes and can be easily adapted to the shape of the bone defect, which facilitates its clinical application. In this research, six formulations of brushite (dicalcium phosphate dihydrated) cement were obtained and the effect of the addition of sodium alginate was analyzed, such as its capacity as a tetracycline release system. The samples that contain sodium alginate set in 4 or 5 min and showed a high percentage of injectability (93%). The cements exhibit compression resistance values between 1.6 and 2.6 MPa. The drug was released in a range between 12.6 and 13.2% after 7 days. The antimicrobial activity of all the cements containing antibiotics was proven. All samples reached values of cell viability above 70 percent. We also observed that the addition of the sodium alginate and tetracycline improved the cell viability.

## 1. Introduction

Calcium phosphates are biomaterials well known to stimulate bone regeneration and have excellent biocompatibility and bioactivity, which has led to a substantial increase in their use in biomedical applications in the last three decades.

These materials are widely used in various applications of orthopedic and maxillofacial surgery, whether for alveolar ridge augmentation, filling of bone defects, middle ear implants, fusion of spinal vertebrae or in the coating of metal prostheses. They are applied in different ways: as granules, blocks or as cements [1]. Due to their excellent biocompatibility, bioactivity and osteoconductivity, they can be reabsorbed by new bone, by the action of bone cells (osteoclasts and osteoblasts) responsible for bone remodeling [2,3]. Since the first research conducted in the 1980s [4], calcium phosphate cements (CPC) have attracted significant interest as a bone substitute. Additionally, due to the malleability of CPC, they have the ability to adapt to bone defects and implant sites, and then harden in situ to provide stability and support [5]. Unlike other materials, these biomaterials can repair bone defects permanently [6], promoting the formation of new bone tissue during cement degradation [7] due to their osteoconductivity [8,9]. In addition, the characteristics of calcium phosphate cements make them an excellent alternative for the release of drugs and other active ingredients, including growth factors and cells [10,11].

Inorganic CPCs often have critical drawbacks that limit their possible clinical application, including a lack of injectability [12,13] that is generally characterized by phase separation during injection, low mechanical properties for the loading requirements of the implantation site [14,15] as well as a weak cohesion that results in the disintegration of the cement paste when in contact with physiological fluids [2,13].

Brushite cements (dicalcium phosphate dihydrated, DCPD, CaHPO_4_·2H_2_O) are prepared by mixing water with a powder consisting of an acid calcium phosphate (monocalcium phosphate monohydrated, MCPM, CaHPO_4_·H_2_O) and a basic calcium phosphate (β-tricalcium phosphate, β-TCP, β-Ca_3_(PO_4_)_2_). The result of this mixture is a moldable paste that solidifies by an exothermic reaction, forming a hard material. These materials were first described in 1989 by Mirtchi and Lemaître [6,16,17].

The first studies performed showed that brushite cements, despite their biocompatibility, are difficult to handle, their setting time is too short (usually less than 30 s) and they have poor mechanical properties [2,12,13,18].

Different additives have been added to these cements to improve some of their properties like injectability [5], cohesion and mechanical properties [19], and the setting time [6]. Polymers have been proven to enhance the mechanical properties of cements due to the role they fulfill in the bone itself, which is a composite material made of an organic phase reinforced with hydroxyapatite crystals. Between the natural polymers, sodium alginate has been studied for many biomedical applications because it is biocompatible, biodegradable and able to form hydrogels. Sodium alginate hydrogels can be prepared under mild conditions by ionic crosslinking and shows a structural similarity to the extracellular matrices of living tissues, which leads to use in applications such as the administration of bioactive agents, the healing of wounds and in tissue engineering [13,20,21,22,23,24,25,26].

Other concerns in maxillofacial surgical operations include the risk of infections that demand the use of antibiotics. A large number of infections in bone implants are caused by bacteria, which are very common in the oral cavity and are related to periodontal diseases. However, antibiotics generally have a negative effect on the mechanical properties of cements due to two effects: the increase in the porosity and the inhibitory effect on the setting reaction [10]. One of the most widely used antibiotics in stomatology is tetracycline, which is known as a very effective antibiotic, with a broad spectrum against bacterial infections, generally related to periodontal diseases [27,28].

In our present study, several brushite or DCPD bone cements for maxillofacial applications made from MCPM and β-TCP with or without sodium alginate were prepared and evaluated as drug release systems for tetracycline. Although the use of CPC as a drug delivery system has been analyzed, the study of more complex formulations that include reinforcement materials, such as sodium alginate, could have a significant impact on the development of more efficient bone regenerative biomaterials with the capability to be used as a drug delivery system and an injectable restoration biomaterial.

## 2. Results

All the cements prepared for our study formed a malleable paste that set in about 2 to 3 min. According to thermochemical studies of calcium phosphate minerals, the dissolution of MCPM in calcium and phosphate ions must be exothermic. In the presence of water, the MCPM tends to hydrolyze in diphosphate and calcium ions following the exothermic reaction of Equation (1). Simultaneously, the exothermic dissolution of β-TCP occurs as a result of its exposure to the acidic medium (Equation (2)). Following the initial dissolution of the reagents, the cement undergoes an increase in pH as a result of the exothermic precipitation of brushite crystals (Equation (3)). Finally, the overall reaction of the cement (Equation (4) = Equation (1) + Equation (2) + 4 × Equation (3)) is exothermic and the brushite cements are usually slightly heated in the final set reaction [6].

Ca(H_2_PO_4_)_2_·H_2_O = Ca^2+^ + 2H_2_PO_4_^−^ + H_2_O (1)

β-Ca_3_(PO_4_)_2_ + 4H^+^ = 3Ca^2+^ + 2H_2_PO_4_^−^ (2)

Ca^2+^ + H_2_PO_4_^−^ +2H_2_O = CaHPO_4_·2H_2_O + H^+^ (3)

β-Ca_3_(PO_4_)_2_ + Ca(H_2_PO_4_)_2_·H_2_O + 7H_2_O = 4CaHPO_4_·2H_2_O (4)

It is known that DCPD is a precursor of hydroxyapatite in aqueous solutions, which is thermodynamically more stable, by a dissolution–reprecipitation mechanism. This compound has a relatively low solubility. Therefore, just the presence of water is not enough to trigger the reprecipitation mechanism. However, the aqueous medium in which the DCPD is immersed contains Ca^2+^ ions. In the presence of Ca^2+^ ions, the process occurs according to Equation (5) [29].

6 CaHPO_4_·2H_2_O + (4 − x) Ca_3_^2+^ = Ca_10−X_(HPO_4_) × (PO_4_)_6−X_(OH)_2−X_ + (10 + x) H_2_O + (8 − 2x) H^+^ (5)

### 2.1. X-ray Diffraction (XRD)

**Figure 1:**
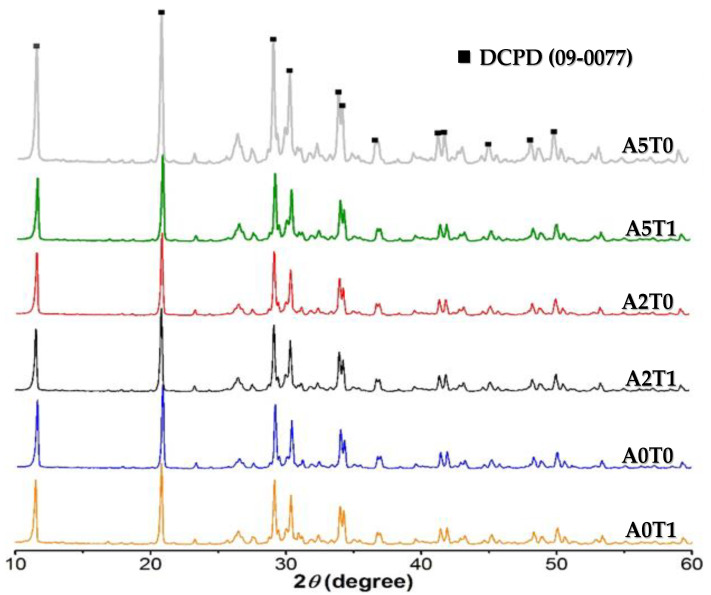
XRD pattern of samples. All peaks correspond to DCPD according to the ICDD PDF 9-0077 X-ray diffraction pattern. The six-maximum intensity DCPD peaks could be observed between 12°and 35° at 2 *θ*.

Figure 1 shows the XRD pattern of the cement samples A0T0, A0T1, A2T0, A2T1, A5T0 and A5T1 after 72 h of immersion in Ringer’s solution. The most significant peaks of the pattern were compared with the XRD pattern of DCPD, which is the expected product of the setting reaction of the cement, using the ICDD PDF 9-0077. A congruence in both position and intensity of the peaks for all samples were observed, which confirms the occurrence of the setting reaction and the precipitation of brushite crystals. It can be observed that the addition of sodium alginate and/or tetracycline do not affect the setting reaction of the cements.

Table 1 shows the crystallite size of the cements. The addition of alginate causes a decrease in the size of the crystals while the incorporation of the drug shows the opposite behavior, but not enough to compensate the effect of the polymer. In none of the cases was there a significant difference between any of the values, whether analyzing the variation of sodium alginate or tetracycline.

### 2.2. Morphology by Electronic Microscopy

#### 2.2.1. Scanning Electronic Microscopy (SEM)

**Figure 2:**
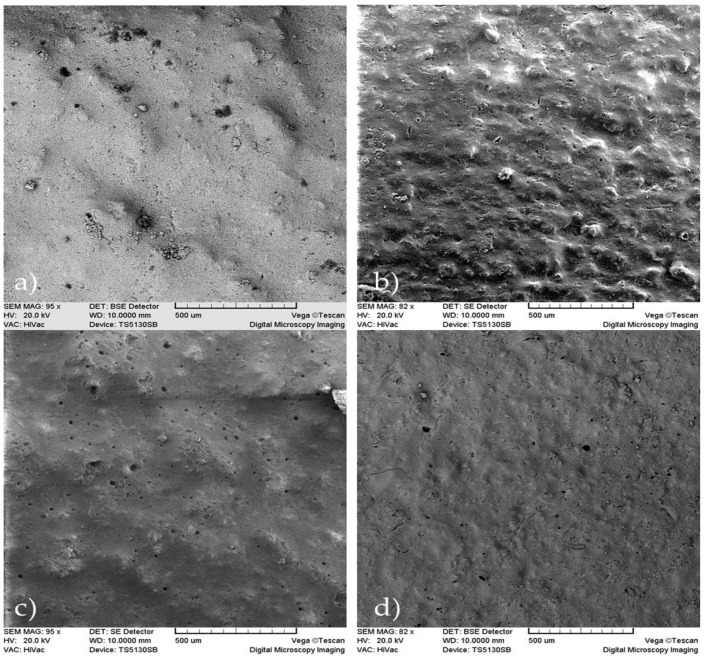
SEM micrographs of the samples: (**a**) A2T0, (**b**) A2T1, (**c**) A5T0 and (**d**) A5T1, (scale bar = 500 µm).

In Figure 2, the micrographs of the samples showed mostly the presence of small size pores, although the presence of macropores can also be observed. In the sample A2T0, we can detect that the presence of alginate causes a low porosity due to its uniform distribution among the calcium phosphate crystals. In A5T0, with the increase of the alginate concentration, the number of pores decreased, although there is a presence of a non-uniform and rough surface that was not observed in the A2T0.

Comparing samples A2T0 and A2T1, it was determined that the presence of tetracycline causes a superficial uniformity, but at the same time there is an increased presence of macropores and deformities that are not observed in the sample lacking tetracycline. When comparing A5T0 with A5T1, a larger roughness of the surface with the presence of macropores is observed in A5T0 that was not formed in A5T1, which is characterized by a more uniform surface and is devoid of macropores.

In A2T1 and A5T1, both samples with tetracycline and different concentrations of alginate, a superficial roughness can be observed as well as a greater number of macropores in A2T1.

#### 2.2.2. Transmission Electronic Microscopy (TEM)

**Figure 3:**
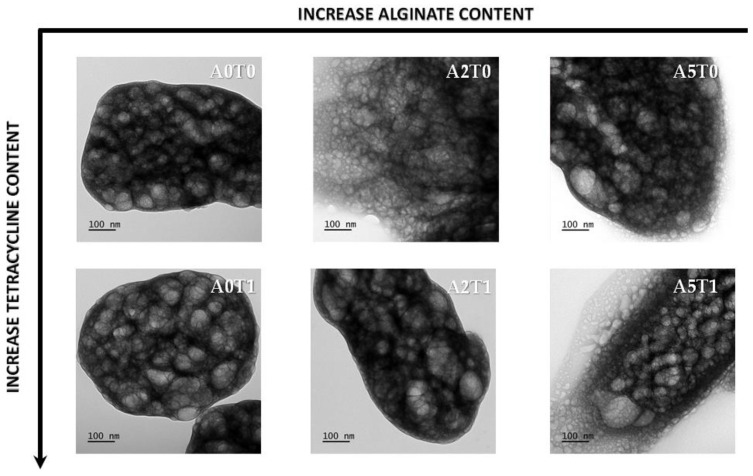
TEM micrographs of all samples at the same magnification.

In Figure 3, the transmission micrographs of the cements show agglomerates of spherical particles of around 50 nm in size. This particle size corresponds with the crystallite size calculated previously (Table 1), and is evidence that increases in the amount of alginate induces a decrease in the size of the crystals. The addition of tetracycline affects the particles size in the opposite way, but the influence is less marked, and cannot counteract the impact of the alginate.

### 2.3. Mechanical Properties

Table 2 shows the results of the mechanical property (compression strength) evaluation. Values between 1.6 and 2.6 MPa for the compressive strength of the samples were obtained, which corresponds to what is reported in the literature for this type of cement [13,16,21,30].

It is also observed that the samples with sodium alginate and without the presence of tetracycline in their formulations (A2T0 and A5T0) were the ones that reached the highest values. As expected, the addition of sodium alginate increases the mechanical properties of the material; this result corresponds with the effect reported in other studies [30].

### 2.4. Injectability

The results of the injectability process are shown in Table 2, where it can be seen that the percentage of injectability ranges between 4.78% and 93.43%. When samples do not contain tetracycline, the addition of sodium alginate does not improve the injectability but makes the cement paste more difficult to handle in the first few minutes and therefore decreases the injectability. In the case of sample A5T0, this effect is more marked by the high viscosity of the liquid phase that does not allow the preparation of the paste in the time required for the test. Thus, the measurement that is reported was made approximately 40 s later than the rest of the samples. In samples with tetracycline, the addition of sodium alginate improved the injectability.

### 2.5. Drug Release Study

In order to study the drug release mechanisms, the Peppas and Sahlin model [31] was used (Equation (6)). In this equation, the first term shows the fraction of drug release. *K*_1_ is the kinetic constant related to the diffusion process and *K*_2_ to the polymer chain relaxation process, and *t* is the time of release. The diffusional coefficient *n* is a measure of the diffusion type for a device of any geometric shape that exhibits a controlled release.

(6) $$\frac{M_{t}}{M_{\infty }}=K_{1}t^{n}+K_{2}t^{2n}$$

**Figure 4:**
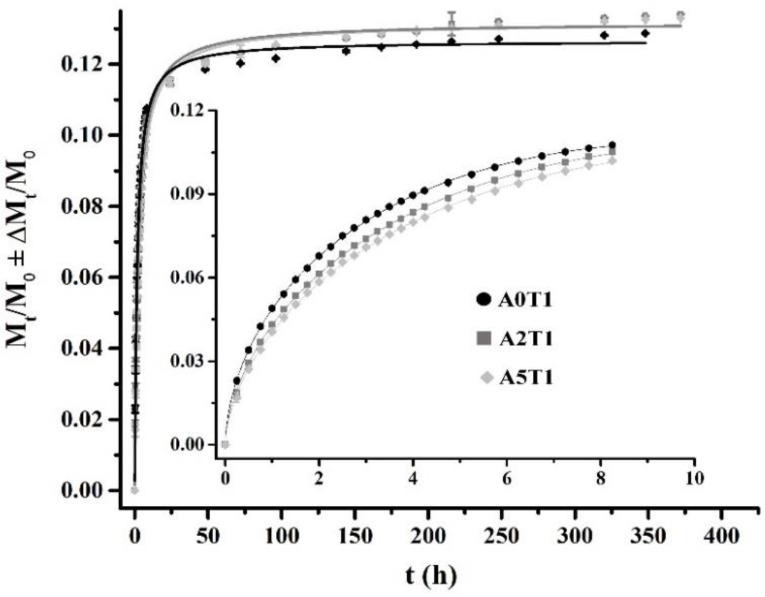
Tetracycline release profiles fitting to Equation (6) (the inset, first 8 h) and Equation (7), from the beginning to the end of the process.

The release profile for the first eight hours (around 75% of the release), as well as the calculated parameters for the Peppas and Sahlin Equation, are shown in Figure 3 and Table 3, respectively. In Figure 4, the inset shows the first eight hours of the release study that is governed by a diffusion mechanism.

It can be observed that as the amount of alginate increases, the system moves away from diffusion (*n* = 0.5) to enter a process where the diffusion governs in conjunction with the relaxation of the polymer chains (*n* > 0.5). The increase in the chain relaxation effect is also described by the increment of *K*_2_, which is the part of the equation associated with the relaxation phenomenon, and the decrease of *K*_1_ that represents the diffusion process (Table 3).

In the inset of Figure 4, it can be seen that the formulation A0T1 showed the highest release in that period of time due to the absence of sodium alginate in the sample, which causes a higher surface porosity and therefore facilitates the release of the drug. However, in the remaining days (full graphic, Figure 3) the other two formulations were the ones that were most released, since A0T1 had already expelled the highest amount of tetracycline in the first stages.

The generalized logistic function or curve, also known as the Richard’s curve, is a mathematical function that appears in various models of population growth, and the spread of epidemic diseases and dissemination in social networks. This function constitutes an extension of the sigmoid function for the growth of one magnitude [32], and is considered one of the best options to adjust dissolution curves [33]:

(7) $$y=A2+\frac{A1-A2}{1+{\left(\frac{x}{x_{0\;}}\right)}^{p}}$$

where *y* is the amount of tetracycline released, *x* is time in minutes, *A*1 is the lower asymptote, *A*2 is the upper asymptote, *x*_0_ is the value of the central node and *p* is the growth rate.

The values of the parameters for each sample, based on the model of Equation (7), can be seen in Table 4. The asymptote *A*1 shows values very close to zero, while the upper asymptote *A*2 shows values that correspond to the maximum percentage of drug released by each sample. In addition, the *p* values for each sample are approximately 0.9. Coefficients of determination with a value greater than 99% were obtained for the fitting of all the release curves, which confirms that this model explains more than 99% of the variability in the amount of drug release.

### 2.6. Microbiological Study

**Figure 5:**
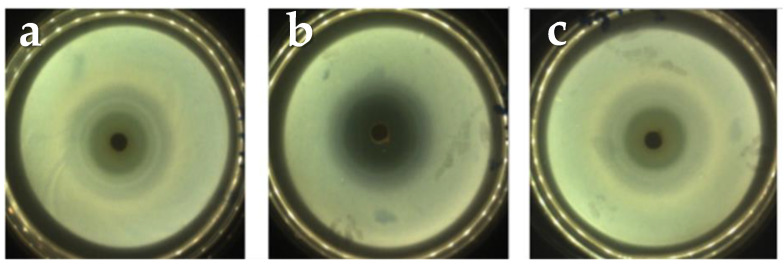
Diameters of the inhibition halos from the A2T1 sample at: (**a**) 24 h, (**b**) 48 h and (**c**) 72 h. The other sample (A5T1) shows a similar behavior.

Figure 5 shows the inhibition zones where no bacterial growth occurred due to the release of the drug to the culture medium.

The measurements of the halos at 24, 48 and 72 h respectively can be observed in Table 5. Here, it is important to notice that the sample A2T1 showed the greatest inhibition halo, which corresponds with the results obtained in the release study.

### 2.7. pH Study

The pH values of the samples over time are shown in Table 6, where it can be observed that in the first minutes of the setting reaction, the pH remains basic but as the reaction progresses the medium acidifies in accordance with Equations (1)–(4). This effect is more significant with the increase in the sodium alginate contents of the formulations.

### 2.8. Cell Viability Test

**Figure 6:**
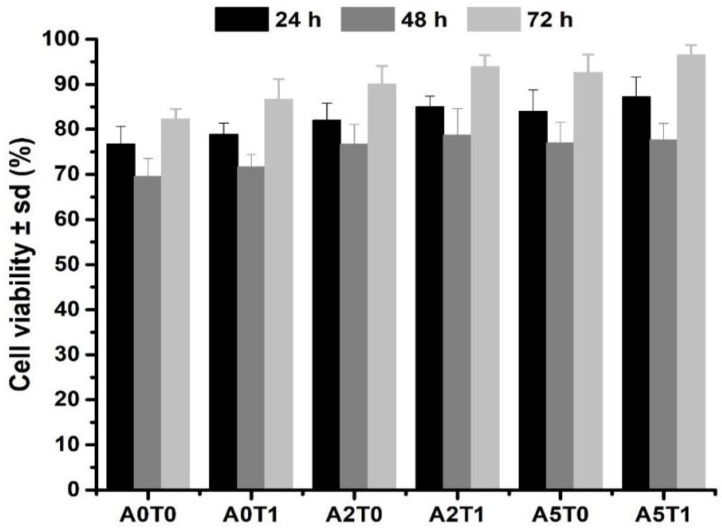
Cell viability assay of the samples at the 24, 48 and 72 h.

Figure 6 displays the cell viability percentages of the samples in the first 72 h of the initial setting. All samples show a percentage above 70, which means that the materials are not cytotoxic according to the standard ISO 10993-5. It also can be seen that the addition of sodium alginate and tetracycline improve the cell viability.

## 3. Discussion

The addition of sodium alginate interferes in the setting reaction, delaying the nucleation of the brushite crystals by making a substitution with the Ca^2+^ ions in the solution. This delay in the initial growth of the crystals causes a marked decrease in the final size of crystallites, as the amount of alginate increases. On the other hand, the presence of the drug also affects the initial growth of the crystals, as explained elsewhere. However, in the presence of alginate, the effect is opposite, but not enough to completely counteract the influence of the drug. The change to a higher value of the cation valence causes an increase in the stability of the alginate complexes that go from being inter- or intramolecular to only being intermolecular at higher valences. Compared with sodium (monovalent cation), the stability of the calcium chelate is remarkably higher than that of sodium, not only for the solubility or viscosity, but several thermodynamic evidences [34,35].

In this study, we showed that the addition of tetracycline in combination with the alginate causes a decrease in the compressive strength of CPC. Tetracycline tends to form chelates with Ca^2+^ ions, which causes a delay in the primary nucleation of the crystals [34,35]. This produces a greater porosity and an increase in setting time, which affects the mechanical properties of cement [10,16,21,23].

The decrease in compressive strength values is expected after 72 h of incubation. The solubility and high hydration capacity of MCPM and the biodegradability of β-TCP are conditioning factors for the low values of mechanical properties, even with the addition of alginate, widely reported and discussed, which should increase them, but competes with the drug’s solubility that decreases it [10,13,21].

In the samples with tetracycline, the presence of alginate improved the injectability. Samples with a drug as a salt are more injectable, which is also evidence that the presence of antibiotics affects the setting reaction of the cement, causing it to be retarded. This allows the material to be more fluid for a longer period of time, promoting injectability, as has been reported in previous investigations [10,12,13].

The release of tetracycline in the matrices is guided by a diffusion mechanism in the first few hours, mainly due to the drug that is close to the edges of the matrix and according to the geometric shapes and the drug solubility. In the case of the samples that contain sodium alginate, the release is controlled by a diffusion mechanism in conjunction with the relaxation of the polymer chains. For a second stage, the release no longer depends only on the solubility of the drug, but also on other factors, such as the advance of the release front, the concentration of the drug and the diffusion medium added. In this case, the logistic function that fits the release profile manages to encompass not only the mechanisms of the second stage but also the diffusion mechanisms that govern the first stage, which is why it is able to describe the entire release process [10,30,32,33,36].

The effectivity of the cements as drug release systems was proven by the inhibition zones, within which there was no bacterial growth in the culture medium. The results of the microbiological study demonstrated that the antibiotic not only is released, but also maintains its pharmacological activity [30,37,38].

The pH study reveals that the increment of hydroxyl groups causes a drop in the pH around the cement due to the presence of alginate. The crosslinking among Ca^2+^ and alginate increases the viscosity of the medium in the first stages. In addition, the Ca^2+^ incorporated in the crosslinking process with alginate releases PO_4_^3−^ into the medium, contributing to the possibility of a decrease of pH values [24,26,39]. This is an important factor to take into account when viability tests of cells are made, since it is a determinant for the survival of the cells in the in vitro tests.

The addition of sodium alginate, a biocompatible natural polymer, provides a substrate that promotes cell growth, resulting in an increase in the cell viability [12,25,26,40]. Moreover, tetracycline creates a safe nest, free from bacteria, which can positively affect the cell development in the implant.

## 4. Materials and Methods

### 4.1. Cement Preparation

All chemicals employed were of analytical grade, used as received. β-TCP was synthetized by a wet neutralization reaction using CaO and H_3_PO_4_ (both: Merck KGaA, Darmstadt, Germany), following the method described by Carrodeguas and de Aza [41].

For the preparation of the CPC, MCPM (Merck KGaA, Darmstadt, Germany) and β-TCP were mixed in the solid phase and, depending on the experiment, 2 or 5% of sodium alginate was added to the liquid phase as a reinforcement. In order to determine their possible uses as a drug release system, 1% tetracycline (Ningxia Qiyuan Pharmaceutical Co., Yinchuan, China) was added as an antibiotic. A solution of sodium citrate was used as a liquid phase of the cements and as a setting retarder. A comparative study to analyze the composition effect on compressive strength, injectability, drug release and antimicrobial activity was carried out. The effect of the independent variables, the quantity of β-TCP used and the addition or not of sodium alginate and/or tetracycline, was studied through the experiments described in Table 7.

### 4.2. X-ray Diffraction (XRD)

Phase characterization was carried out by means of X-ray diffraction, in a Rigaku Rotaflex, RU200B diffractometer with Cu-K_α_ radiation (1.54056 nm). The scans were made in a 2 θ angular interval of 10–60° and a scanning speed of 0.02°/min. The results were interpreted using the X’Pert HighScore PANalytical program database, version 3.0 (PANalytical B. V. Almelo, The Netherlands). Crystal size was calculated using the Debye Scherrer tools of the software.

### 4.3. Electronic Microscopy

#### 4.3.1. Scanning Electronic Microscopy (SEM)

The samples were coated with a 20 nm film of gold in a BAL-TEC MED 020 system and placed in a desiccator until analysis. A JEOL microscope, JSM-6360LV (Jeol Ltd., Tokyo, Japan) with Oxford EDX probe (Oxford Instruments, High Wycombe, UK), magnification of 5–300,000, resolution of 3 nm and acceleration voltage of 30 kV, was used for the microstructural analysis.

#### 4.3.2. Transmission Electron Microscopy (TEM)

Samples were diluted in Milli-Q water. Subsequently, carbon-coated grids (Formvar/Carbon on 200 Mesh Copper; AGS162; Van Loenen Instruments; Zaandam, the Netherlands) were glow-discharged using the Emitech K950X Turbo Evaporator (Quorum Technologies; Ashford, UK) at 2 × 20^−1^ mbar and 20 mA for 1 min. Next, 3 µL of sample solution were applied on the freshly glow-discharged grid and allowed to adhere for 1 min. Afterwards, excess liquid was discarded by blotting onto a filter paper and the sample was air-dried for 10 min. Grids were mounted on a room temperature holder and examined using a FEI T12 Spirit BioTwin (FEI Company; Hillsboro, OR, USA) equipped with an OneView Camera Model 1095 (Gatan; Pleasanton, CA, USA) at a voltage of 120 kV. Digital images were acquired and stored using DigitalMicrograph 3.4 (Gatan, Pleasanton, CA, USA).

### 4.4. Mechanical Characterization

For the compressive strength of the material, 12 mm height and 6 mm diameter specimens were prepared. The samples were immersed in Ringer’s solution at 37 °C and tested after 24 h of the cement preparation, immediately after being extracted in order to maintain hydration. The study was carried out in a universal testing machine (TestCom-5, IBERTEST, Madrid, Spain) with a load cell of 200 N and at 1 mm min^−1^ load application speed. The compressive strength (σ_c_) in MPa was determined by the following formula:

(8) $$\sigma_{c}=\frac{F}{A_{0}}=\frac{4P}{{\pi d}^{2}}\cdot 10^{-6}$$

where P is the maximum breaking load (N) and d is the diameter of the specimen (m). Five specimens were tested for each formulation.

### 4.5. Injectability Study

The injectability of the samples was determined by extruding a certain quantity of the paste placed in a commercial plastic syringe of 5 mL capacity and with an exit diameter in the nozzle of 2 mm [42]. The extrusion was performed by placing the syringe in a universal testing machine (TestCom-5, IBERTEST, Madrid, Spain) using a compression speed of 15 mm/min until reaching a maximum load of 100 N [43]:

(9) $$\%Injectability=\frac{mass\;of\;injected\;material}{total\;mass\;of\;material}\cdot 100\%$$

### 4.6. Drug Release Study

Test specimens of the cements of 6 mm in height and 12 mm in diameter loaded with tetracycline were used. The samples were immersed in 10 mL of Ringer’s solution in glass bottles at (37.0 ± 0.5) °C throughout the study. The solution in contact with the specimens was completely extracted at the established times and replaced with 10 mL of fresh solution. The extractions were made every half hour until 5 h of the cement preparation and, after that, at 24 h up to seven days. Five specimens of each formulation were prepared and evaluated. The determination of the antibiotic released to the solution was carried out in a UV-Visible Spectrophotometer (Shimadzu, Kyoto, Japan) at a wavelength of 276 nm and the results were reported as a cumulative amount of the tetracycline released versus time [44].

### 4.7. Microbiological Study

For the microbiological study, specimens of 6 mm in height and 12 mm in diameter were prepared. In order to evaluate the antimicrobial susceptibility of the composites, strains of Staphylococcus aureus Agar Tripton were used, at a strain concentration adjusted with a turbidimetric method employing as a reference a 0.5 MacFarland standard (1 × 10^8^ CFU mL^−1^). Subsequently, 500 µL of a previously prepared culture medium of Mueller-Hinton agar (Merck KGaA, Darmstadt, Germany) was inoculated in Petri dishes. After a period of 20 min the test specimens were placed on top of the plates containing the culture medium and the bacterial suspension and were incubated at 37 ± 1 °C for a period of 72 h. Three specimens were tested for each formulation and the inhibition zone was measured with the software SCAN 500 Automatic Colony Counter Version 6.

### 4.8. pH Study

For the pH study, the samples of 300 mg approximately, were immersed in 10 mL of PBS at 37 °C of temperature and the pH was measure (HI-83300 pH-meter, Hanna Instruments, Woonsocket, RI, USA) over 7 h of the first day and then at the 24 and 96 h.

### 4.9. Cell Viability

MTS assay. To further corroborate the cell viability results, an MTS assay was performed. This is a colorimetric technique in which (3-(4,5-dimethylthiazol-2-yl)-5-(3-carboxymethoxyphenyl)-2-(4-sulfophenyl)-2H-tetrazolium), in the presence of phenazine methosulfate (PMS), produces a formazan product that has an absorbance maximum at 490 nm in PBS. Scaffold samples with dimensions like a viability assay were loaded with osteoblastic MC3T3-E1 cells (density: 10^4^ per well; 500 µL of cell suspension), and then incubated for 24, 48 and 72 h; 100 µL of the supernatant solution was extracted to a 96-well plate for reading into a tunable, spectrophotometric microplate reader (VersaMax, Molecular Devices, San José, CA, USA with Program Softmax Pro) and the absorbance (λ = 490 nm) was measured.

### 4.10. Statistical Calculations

Graphs and statistics were performed with OriginPro 2021 (OriginLab Corp., Northampton, MA, USA). Data are reported as mean ± standard deviation (SD), unless stated otherwise. Error bars represent the SD calculated from tests of triplicate measurements for each scaffold.

## 5. Conclusions

Six DCPD formulations were obtained in which the influence of the addition of sodium alginate and/or tetracycline on magnitudes such as mechanical properties, release capacity, injectability, microbiological response and cell proliferation was studied. The addition of sodium alginate caused an increase in mechanical properties and cell proliferation, as well as release in the final stages. The injectability and the pH values decreased, as well the release in the first stage, due to its dependence on diffusion and where the viscosity provided by the sodium alginate interferes with the process.

The addition of tetracycline had less marked effects. In this case, all the magnitudes increased their value except the case of pH, results that agree with the state of the art of the subject. Certainly, when both substances coincided in the formulation, the values of the properties broke the trend, an unequivocal sign of materials science, where magnifying one property leads to sacrificing the values of another. However, the materials obtained proved to be a promising option in the restoration of bone tissue with the added functionality of a controlled drug release system.

## Figures and Tables

**Table 1:** Crystallite size of the cements.

Samples	A0T0	A0T1	A2T0	A2T1	A5T0	A5T1
Crystallite Size (nm)	58 ± 7	57 ± 7	54 ± 4	56 ± 7	50 ± 4	58 ± 7

**Table 2:** Compression strength (after 72 h of incubation) and injectability of the samples.

Samples	Compressive Strength (MPa)	Injectability (%)
A0T0	1.6 ± 0.5	72.56
A0T1	1.9 ± 0.3	87.07
A2T0	2.6 ± 0.2	41.71
A2T1	1.9 ± 0.5	93.04
A5T0	2.3 ± 0.7	4.78
A5T1	1.9 ± 0.4	93.43

**Table 3:** Values of the parameters of the Peppas and Sahlin Equation for each sample (inset Figure 3, first eight hours).

Plot	A0T1	A2T1	A5T1
*K*_1_	0.0559 ± 0.0001	0.0483 ± 0.0002	0.0452 ± 0.0002
*K*_2_	−0.00719 ± 0.00004	−0.00537 ± 0.00005	−0.00482 ± 0.00006
*n*	0.589 ± 0.003	0.610 ± 0.004	0.620 ± 0.006
*R* ^2^	99.99%	99.98%	99.97%

**Table 4:** Values of the parameters of the logistic curve for each sample (full graphic, Figure 4, 15 days).

Plot	A0T1	A2T1	A5T1
*A*1	0.003 ± 0.002	0.00162 ± 0.00189	0.00142 ± 0.00144
*A*2	0.1266 ± 0.0008	0.1319 ± 0.0008	0.1319 ± 0.0006
*x*_0_	1.70 ± 0.07	2.27 ± 0.09	2.54 ± 0.08
*p*	0.98 ± 0.03	0.93 ± 0.03	0.92 ± 0.02
*R* ^2^	99.58%	99.65%	99.80%

**Table 5:** Diameter of the growth inhibition halos (mm) on time.

Samples	24 h	48 h	72 h
A0T1	37 ± 2	37 ± 2	37 ± 2
A2T1	37.0 ± 0.6	38 ± 1	40.1 ± 0.5
A5T1	37.5 ± 0.5	37.6 ± 0.4	37.7 ± 0.3

**Table 6:** Investigation of pH values of the samples.

t (min)	t (h)	A0T0	A0T1	A2T0	A2T1	A5T0	A5T1
5	0.08	7.24	7.24	7.26	7.22	7.19	7.23
60	1	7.06	6.87	7.13	7.16	7.11	7.13
120	2	6.92	6.37	7.01	6.69	6.84	6.54
185	3	6.84	6.28	6.79	6.63	6.35	6.42
240	4	6.78	5.93	6.74	6.44	5.98	6.33
300	5	6.74	5.81	6.50	6.22	5.70	6.17
360	6	6.72	5.71	6.41	6.18	5.58	6.12
420	7	6.67	5.68	6.36	6.10	5.49	6.08
1440	24	6.60	5.41	6.29	5.89	4.91	5.87
2304	96	6.47	5.01	6.05	5.60	4.74	5.45

**Table 7:** Experimental design, MCPM/β-TCP = 45/55%. Liquid phase = 0.5 mL/g.

Series	Sodium Alginate (% *w*/*w*)	Tetracycline (% *w*/*w*)
A0T0	0	0
A0T1	0	1
A2T0	2	0
A2T1	2	1
A5T0	5	0
A5T1	5	1

## Data Availability

The data presented in this study are available on request from the corresponding author.
